# From Polymer Structure to Valve Function: A Multiscale Evaluation of Polycarbonate Polyurethanes for Polymeric Mitral Valves

**DOI:** 10.1007/s10439-026-04308-1

**Published:** 2026-08-06

**Authors:** Farhad Sadeghi, Nnaoma Agwu, Mia Tacklind, Arash Kheradvar

**Affiliations:** 1 Department of Biomedical Engineering, University of California, 2420 Engineering Hall, Irvine, CA 92697-2730, USA; 2 Department of Radiological Sciences, University of California, 101 The City Drive South, Orange, Irvine 92868-3298, USA

**Keywords:** Polymeric heart valve, Mitral valve, Particle image velocimetry, Accelerated wear testing, Hemodynamic performance, Hybrid tissue-engineered heart valve

## Abstract

**Purpose:**

Polymeric heart valves (PHVs) offer the potential to combine the durability of mechanical valves with the favorable hemodynamics of bioprosthetic valves; however, establishing clear relationships between polymer properties and valve-level function remains a key challenge. This study evaluates polycarbonate-based polyurethanes as candidate scaffold materials for polymeric mitral valves through a multiscale structure–function approach.

**Methods:**

Two aromatic polycarbonate-based thermoplastic polyurethanes, Carbothane AC-4095A (CB95-AC) and QuadraSil ARCS 90A (Qsil), were characterized using rheological, mechanical, and spectroscopic analyses. Trileaflet valve scaffolds were fabricated and evaluated using particle image velocimetry (PIV), pressure–flow measurements, and accelerated wear testing (AWT) up to 50 million cycles. Preliminary *in vivo* performance was assessed following implantation of a CB95-AC valve in an ovine model.

**Results:**

Qsil exhibited higher solution viscosity, lower storage modulus, and improved elastic recovery, indicating a more compliant response, whereas CB95-AC demonstrated greater stiffness and tear resistance. Both materials maintained stable valve function, with preserved projected orifice area and repeatable pressure waveforms after AWT. PIV analysis showed physiologic pulsatile inflow and intraventricular vortex formation for both valves, with Qsil producing slightly broader vorticity patterns and CB95-AC exhibiting more localized flow structures. Evaluation of the CB95-AC valve *in vivo* demonstrated preserved transmitral inflow with low pressure gradients and no evidence of obstruction.

**Conclusion:**

Polycarbonate-based polyurethanes provide promising performance as polymeric mitral valve scaffolds, with material-dependent differences influencing leaflet mechanics and flow organization while maintaining structural integrity during comparative accelerated wear testing. These findings provide a multiscale framework for evaluating associations between polymer properties, mechanical behavior, and valve-level performance in next-generation polymeric and hybrid tissue-engineered heart valves.

## Introduction

Heart valve replacement remains the standard treatment for severe valvular disease, with current prosthetic options limited to mechanical heart valves (MHVs) and bioprosthetic heart valves (BHVs). MHVs, typically made from rigid materials such as pyrolytic carbon, provide excellent durability but require lifelong anticoagulation due to thromboembolic risk [1]. In contrast, BHVs offer improved hemodynamics and lower thrombogenicity but suffer from structural degeneration and limited durability, particularly in younger patients [2], [3]. Polymeric heart valves (PHVs) have emerged as a promising alternative, aiming to bridge this gap by combining the durability of mechanical valves with the favorable hemodynamic and biocompatible properties of tissue valves [4].

Recent advances in polymer chemistry have improved the mechanical performance and hemocompatibility of PHVs; however, challenges such as calcification, thrombosis, limited bioactivity, and long-term structural stability remain [5–8].

These limitations have motivated the development of hybrid tissue-engineered heart valves (H-TEHVs), [9, 10 ] which integrate a durable synthetic scaffold with biologic components to promote in situ remodeling while maintaining mechanical integrity [11]. In this context, the design of the polymeric scaffold is critical, as its molecular structure governs mechanical response, fatigue resistance, and ultimately valve-level function [12, 13 ], [14]. As a result, a comprehensive framework connecting material composition to valve function—particularly under physiologic mitral flow conditions—remains lacking [15], [16].

To address this gap, a multiscale evaluation strategy is required. At the material level, the microphase structure of segmented thermoplastic polyurethanes (TPUs), governed by hard–soft segment interactions, determines stiffness, elasticity, and resistance to fatigue. At the device level, these properties directly influence leaflet deformation, coaptation, and dynamic response during cyclic loading. At the functional level, valve performance must be assessed through physiologically relevant metrics, including intraventricular flow patterns and long-term durability. Particle image velocimetry (PIV) provides high-resolution characterization of flow structures such as vortex formation and shear distribution, which are critical determinants of thrombogenicity and ventricular efficiency [12], [13]. Complementarily, accelerated wear testing (AWT) enables evaluation of structural integrity and functional stability under prolonged cyclic loading conditions consistent with ISO 5840 standards [14].

Building on our previous identification of polycarbonate-based polyurethanes as promising biomaterials [17], the present study systematically investigates how differences in polymer composition influence valve mechanics, durability, and hemodynamic function. Specifically, two medical-grade aromatic TPUs were investigated: a polycarbonate polyurethane, Carbothane AC-4095A, and a polycarbonate–siloxane copolyurethane QuadraSil ARCS 90A were evaluated as candidate scaffold materials for polymeric mitral valves. Material properties were characterized through rheological, mechanical, and spectroscopic analyses, while valve-level performance was assessed using PIV, AWT, and pressure flow measurements under physiologic conditions. In addition, preliminary *in vivo* evaluation was performed following implantation of a CB95-AC valve in an ovine model. By integrating material characterization with functional valve assessment, this work investigates relationships between polymer composition, mechanical behavior, and valve-level function across multiple length scales. While the study identifies consistent associations between material properties and valve performance, it is not intended to establish a direct mechanistic chemistry-to-function pathway.

## Materials and Methods

### Materials Preparation and Characterization

**Fig. 1:**
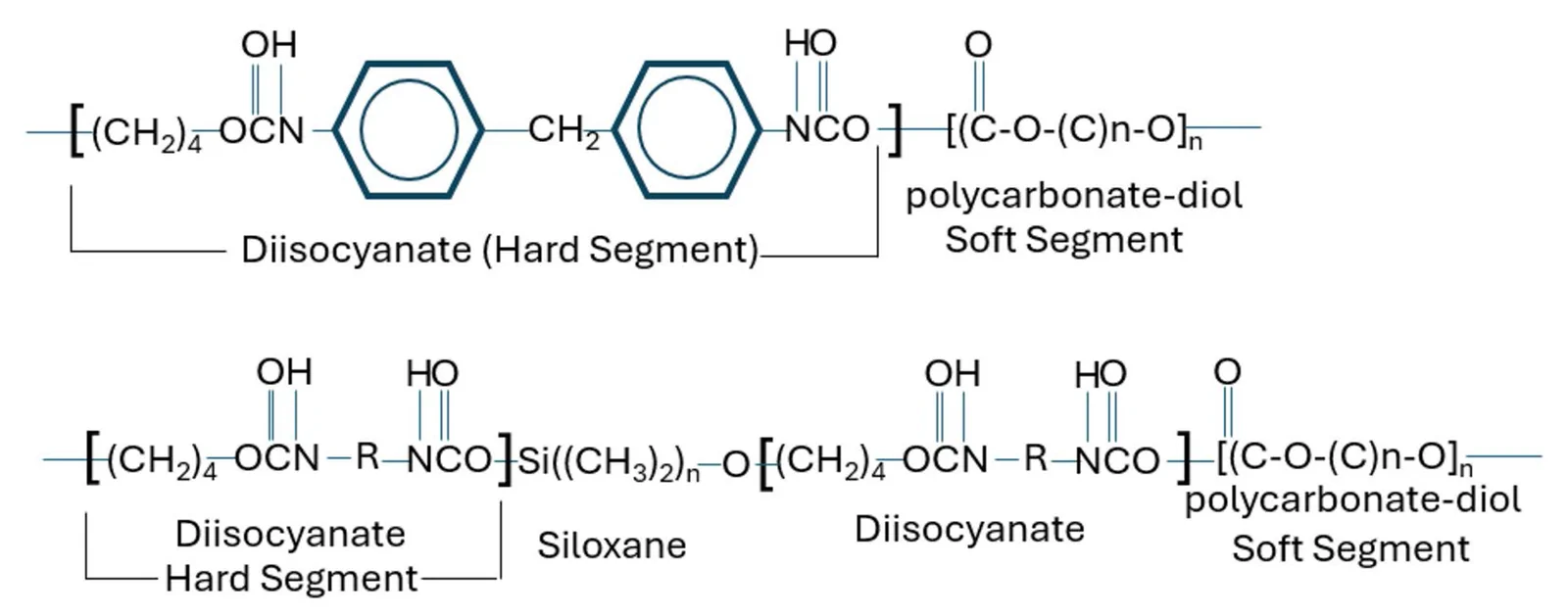
Representative molecular architectures of polycarbonate-based and polysiloxane-modified thermoplastic polyurethanes (TPUs): 1) isocyanate, 2) polyol, which may be polyester, polyether, or polycarbonate, and 3) a chain extender. The reaction of these components forms a morphology known as a segmented structure, including alternating soft and hard segments. The combination of the chain extender and diisocyanate forms the hard segments, while the polyol component forms the soft segments

Two aromatic polycarbonate-based thermoplastic polyurethanes (PC-TPUs) were studied: Carbothane AC-4095A (Lubrizol) and QuadraSil ARCS 90A (Biomerics), hereafter referred to as CB95-AC and Qsil, respectively. Both materials are segmented polyurethanes composed of hard and soft domains, where the relative composition and intermolecular interactions govern mechanical and viscoelastic behavior (Fig. 1).

The polymer selection in the present study reflects the distinct mechanical requirements of a flexible valve leaflet compared with a tissue-engineering mesh or scaffold. Although ChronoFlex 65D showed promise in our previous study for cell-supportive scaffold applications, its higher stiffness limited leaflet deformation under cyclic loading. Therefore, CB95-AC and Qsil were selected in this study as more compliant candidate materials for mitral leaflet fabrication [17].

Polymer solutions (20 wt%) were prepared by dissolving resin granules in dimethylacetamide (DMAc) at 60 °C for 24 h. For material characterization, two-layer films were fabricated by sequential solution casting, with each coating layer dried at 60 °C and having a thickness of approximately 80–90 μm. Rheological properties were measured using a Discovery Series Hybrid Rheometer HR-2 (TA Instruments) under oscillatory conditions at room temperature.

Dynamic mechanical analysis (DMA Q800, TA Instruments, New Castle, DE) was performed in tension mode with 0.1% strain to assess stiffness and viscoelastic behavior.

Thermal properties of the films were analyzed using a differential scanning calorimeter (DSC 2500) (TA Instruments, New Castle, DE). The samples were heated from − 70 °C to 200 °C at a rate of 10 °C/min, cooled at the same rate to room temperature, and then reheated to 200 °C.

Mechanical properties were evaluated using an Instron 3365 testing system. Tensile and cyclic hysteresis tests were performed at a crosshead speed of 100 mm/min using specimens with a gauge length of 25 mm and a width of 15 mm. Tear resistance was measured using notched specimens with a width of 25 mm at a crosshead speed of 500 mm/min, and the maximum force at failure was recorded. All tensile and tear tests were performed in triplicate. For tensile testing, representative stress–strain curves are presented, while tear resistance results are reported as mean ± standard deviation. To assess oxidative stability, samples were subjected to accelerated aging in 0.1 M CoCl_2_ and 20% H_2_O_2_ at room temperature for 30 days. [18].

Chemical structure and microphase organization were analyzed using Fourier transform infrared spectroscopy (FTIR; Nicolet Apex, Thermo Fisher Scientific). The ratio of hydrogen bonded to free carbonyl groups was estimated from the absorbance peaks near ~ 1700 cm^−1^ and ~ 1735 cm^−1^, providing an indicator of hard-segment association and phase separation [19].

To evaluate the effect of H_2_O_2_ exposure on the carbonyl environment, the ATR-FTIR carbonyl region (approximately 1770–1660 cm^−^1) was analyzed by comparing the free-to-hydrogen-bonded carbonyl peak-area ratio, calculated as (A1735)/(A(1722) + A(1700)), between unaged and H_2_O_2_-aged samples. Changes in this ratio were interpreted as changes in the relative carbonyl hydrogen bonding environment and hard-segment organization following oxidative exposure, rather than as direct evidence of microphase morphology or oxidative degradation [20].

The silicon content of the TPU formulations was evaluated by energy-dispersive X-ray fluorescence spectroscopy (EDXRF; EDX-7000, Shimadzu Corporation, Kyoto, Japan) under air atmosphere using a Rh target, 10 mm collimator. Measurements were acquired under automatic conditions at 50 kV for the Ti–U range and 15 kV for the Na–Sc/S–Ca ranges, with 120 s live time per condition, and silicon concentration was quantified using the fundamental-parameters method. Three independent measurements were collected for each sample, and results are reported as mean ± standard deviation (n = 3).

### Valve Casting

Polymeric trileaflet valve scaffolds (27 mm diameter; ~ 190 μm average leaflet thickness) were developed by dip molding in the polymer solutions. Valve prototypes were used for functional and durability assessments. The trileaflet design was selected as a simplified, symmetric prototype to facilitate fabrication and reproducible leaflet motion. Leaflet reinforcement was achieved using a Nitinol wireframe/stent to provide structural support and improve leaflet stability under cyclic loading. Fabrication is an important factor in polymeric valve performance because processing conditions can influence chain organization, residual stress, surface morphology, and leaflet-scale mechanical behavior. In this study, CB95-AC and Qsil were processed using the same dip-molding procedure from 20% DMAc solution, with a dipping speed of 15 mm/min, 30 s dwell time, and withdrawal speed of 15 mm/min, to allow a controlled comparison between formulations. Leaflet thickness was measured using a micrometer to ensure comparable thickness between valve samples. GC-MS headspace analysis of both cast films and fabricated valve samples showed no detectable residual DMAc under the applied conditions. For the animal study, a PET fabric sewing ring was incorporated to facilitate suturing, improve annular fixation, and provide a stable base for surgical implantation.

### Hemodynamic Evaluation and Durability Testing

Valve-level performance was evaluated through particle image velocimetry (PIV), pressure flow measurements, and accelerated wear testing (AWT) under physiologic pulsatile conditions.

Hemodynamic testing was conducted in a pulsatile heartflow simulator (Vivitro SuperPump system) incorporating a compliant silicone left ventricular (LV) sac housed within a pressurized chamber to reproduce physiologicmitral flow conditions [21]. The ventricular model was designed to mimic physiologic geometry and compliance, enabling realistic pulsatile flow generation. Pressure and flow rate were continuously monitored using Deltran-2 pressure transducers (Utah Medical) and transonic flow probes, respectively, with synchronized acquisition of pressure, flow, and piston displacement signals. PIV measurements were performed using a high-speed digital imaging system (Galileo 5 K Veloce, Integrated Design Tools) integrated with a laser-based illumination setup. The flow was seeded with neutrally buoyant tracer particles (typically 15–80 μm diameter) to enable velocity field reconstruction. A pulsed Nd:YLF green laser (Evolution 30, Coherent Inc., Santa Clara, CA) was used to generate a thin laser sheet aligned with the mid-valve plane, effectively sectioning the ventricular model and valve leaflets into a two-dimensional measurement plane. Image acquisition was synchronized with the pulsatile cycle using a BNC 565 pulse delay generator (Berkeley Nucleonics Corporation), ensuring phase-locked measurements at defined time points within the cardiac cycle.

High-speed image sequences were acquired using a high-resolution digital camera system. In this study, we used a Galileo 5 K Veloce, providing high spatial resolution (up to 5120 × 3456 pixels) with frame rates at 1 kHz, enabling accurate capture of rapid intracardiac flow dynamics. The camera was positioned perpendicular to the imaging plane to capture particle motion within the LV chamber.

PIVlab in MATLAB R2025 was used to process and analyze the PIV images. Velocity fields were computed by cross-correlation of sequential image pairs (typical interrogation window size ~ 32 × 32 pixels with 50% overlap), yielding time-resolved velocity vector fields without cycle averaging. From these data, derived flow quantities such as velocity magnitude and vorticity were obtained to characterize transmitral jet development, vortex formation, and intraventricular flow organization during diastole.

Pressure flow characteristics were evaluated concurrently to assess valve opening and closing dynamics. Pulsatile inflow and outflow waveforms, along with transvalvular pressure gradients, were analyzed to determine peak flow rates, timing of valve opening and closure, and overall cyclic hemodynamic behavior.

The accelerated cyclic wear test was performed using a Dynatek Labs M6 Heart Valve Durability Tester to compare the relative durability and cyclic response of CB95-AC and Qsil valves under identical test conditions. The system was operated at 800 cycles/min with a nominal system pressure setting of 140 mmHg at room temperature (25 °C) using distilled water. In this comparative configuration, the peak measured differential pressure was approximately 25–30 mmHg; therefore, the test was used as a material-comparison durability screen rather than a formal ISO 5840 qualification study. The primary objective was to characterize and compare the cyclic response of the two valve materials under the same testing environment.

Valve performance was evaluated before and after testing by visual inspection and image-based assessment of leaflet motion, coaptation, deformation, tearing, delamination, and projected orifice area. The projected orifice area was quantified from high-resolution AWT camera images captured when the valve reached the fully open position during the cycle. Images were imported into ImageJ (NIH, Bethesda, MD, USA) and calibrated using a known reference dimension within the valve fixture. The open flow region bounded by the leaflet free edges was manually outlined, and the enclosed area was calculated using the ImageJ area measurement tool. Three independent measurements were performed for each valve design, and projected orifice area was reported as mean ± standard deviation. This image-based analysis was used to compare relative valve opening characteristics and does not represent the effective orifice area (EOA) determined from hydrodynamic testing.

### In Vivo Evaluation

The in vivo evaluation consisted of an acute implantation study in a 43 kg ovine model, performed under the approved UCI IACUC protocol AUP-25-109, to assess procedural feasibility and immediate valve function. The procedure was conducted under general anesthesia and cardiopulmonary bypass. A single CB95-AC mitral valve prosthesis was implanted using a PET suture ring to facilitate surgical handling and annular fixation. Valve sizing was selected based on the native annular dimensions, and the valve platform was designed to accommodate three valve sizes to support surgical annular matching.

Systemic anticoagulation with heparin was administered during the procedure. Valve performance was evaluated intraoperatively immediately after implantation using an EPIQ CVx ultrasound system (Philips Ultrasound). Transmitral inflow and leaflet function were assessed using color and spectral Doppler echocardiography. The animal remained hemodynamically stable throughout the procedure, and the study endpoint was acute assessment of implantability, leaflet motion, transvalvular flow, and immediate valve function. Since only a single CB95-AC valve was evaluated in this acute animal model, the in vivo findings are presented as an initial feasibility assessment rather than a long-term performance evaluation or comparative assessment of the Qsil valve.

Measured parameters included peak early (E-wave) and atrial (A-wave) filling velocities, E/A ratio, transvalvular pressure gradients, deceleration time, pressure half-time, and calculated mitral valve area. Color Doppler imaging was used to qualitatively evaluate flow patterns and detect potential turbulence or regurgitation.

## Results

### Mechanical and Rheological Characterization

**Fig. 2:**
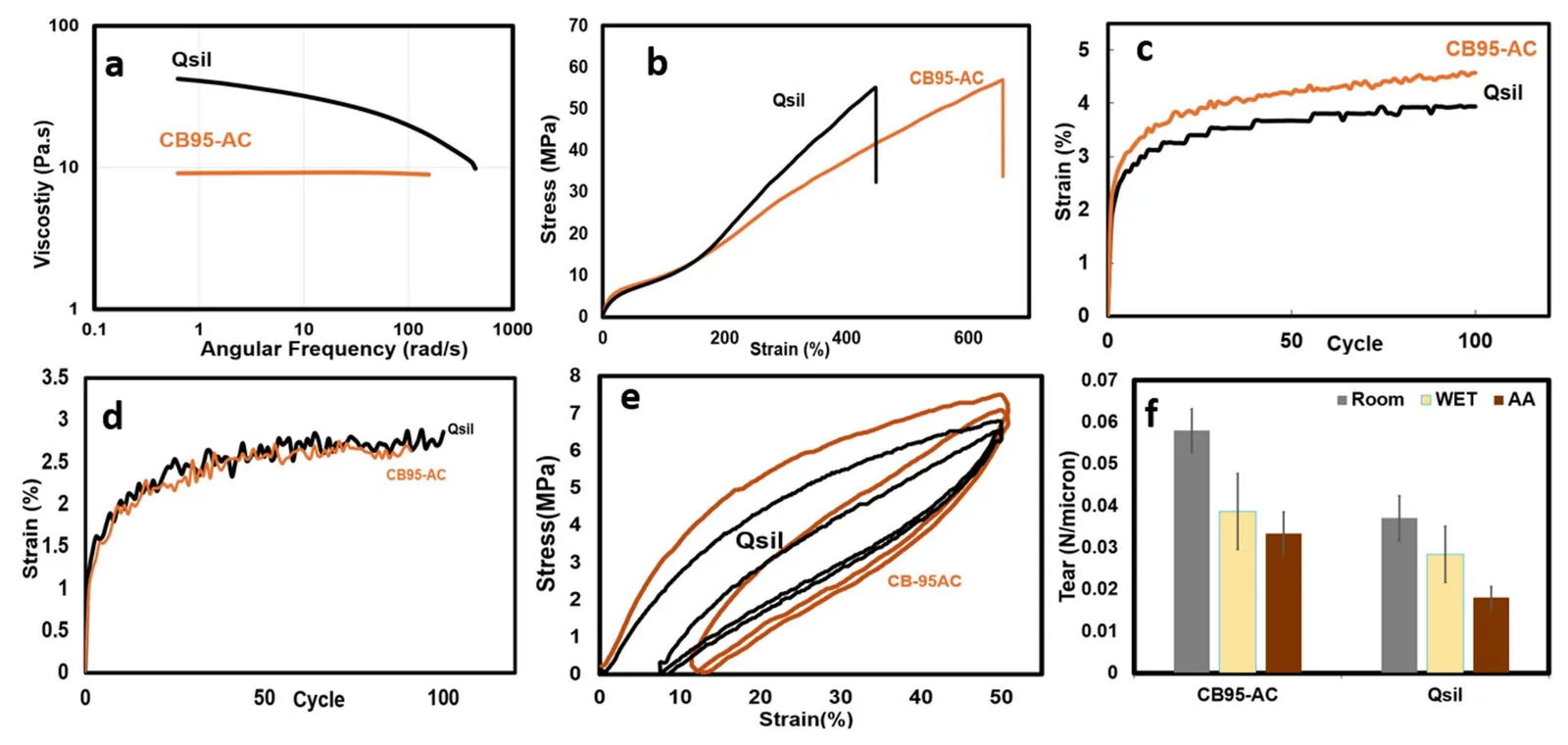
Oscillatory viscometry and mechanical characterization of TPU films. **a** Viscosity–frequency sweep of polymer solutions in DMAc (20% wt) measured at room temperature (25 °C). Qsil exhibited non-Newtonian shear-thinning behavior, with viscosity decreasing as frequency increased, whereas CB95-AC showed substantially lower solution viscosity and behaved nearly as a Newtonian fluid under the same conditions. (**b**–**f**) Mechanical characterization of TPU films. **b** Tensile testing showed that CB95-AC exhibited slightly higher tensile stress during the early stage of deformation; however, the overall tensile responses of CB95-AC and Qsil were comparable. **c** Cyclic hysteresis testing at room temperature showed that Qsil provided improved strain recovery, while all materials reached a more stable cyclic response after approximately 100 cycles. **d** Under wet cyclic loading at 37 °C, both polymers exhibited increased elasticity, lower residual strain, and similar cyclic behavior, although some softening was observed. **e** At large strain (50%), Qsil retained the lowest residual strain, whereas CB95-AC showed greater permanent deformation. **f** Tear resistance was lower for Qsil, and the same trend was observed under wet/37 °C and accelerated-aging conditions

The viscosity–frequency behavior of the 20% (wt) polyurethane solutions in DMAc is shown in Fig. 2a. Qsil exhibited consistently higher viscosity across the tested frequency range and demonstrated pronounced shear-thinning behavior, indicating a more non-Newtonian response. In contrast, CB95-AC showed lower viscosity with minimal frequency dependence, consistent with near-Newtonian behavior.

EDX analysis confirmed the presence of silicon in Qsil, with an average silicon content of 26.7 ± 0.15 wt% (n = 3), while no detectable silicon was observed in CB95-AC. The measured silicon content is consistent with the incorporation of a siloxane-containing phase within the Qsil formulation and provides direct compositional evidence distinguishing Qsil from the conventional polyurethane control.

Tensile stress–strain results (Fig. 2b) revealed that CB95-AC exhibited slightly higher stress at low strain, suggesting greater initial stiffness. At higher strain levels, Qsil demonstrated stronger strain hardening and reached comparable or slightly higher maximum stress, while CB95-AC exhibited greater elongation prior to failure.

Cyclic hysteresis testing (Fig. 2c) showed that Qsil exhibited lower residual strain and improved elastic recovery compared to CB95-AC. For both materials, residual strain increased rapidly during early cycles and gradually approached a plateau over approximately 100 cycles. Under physiologic conditions (wet, 37 °C; Fig. 2d), both materials showed similar cyclic response behavior, with lower residual strain compared with the room-temperature condition. Under large deformation (50% strain; Fig. 2e), Qsil exhibited lower residual strain compared with CB95-AC, indicating improved recovery under high-strain loading conditions. Tear resistance results (Fig. 2f) showed that CB95-AC consistently exhibited higher tear strength than Qsil under all tested conditions (room temperature, wet, and accelerated aging). Both materials demonstrated reduced tear resistance following moisture exposure and oxidative aging, although the relative ranking remained unchanged, with CB95-AC maintaining superior resistance to tear propagation.

### Chemical Characterization and Structure

**Fig.3:**
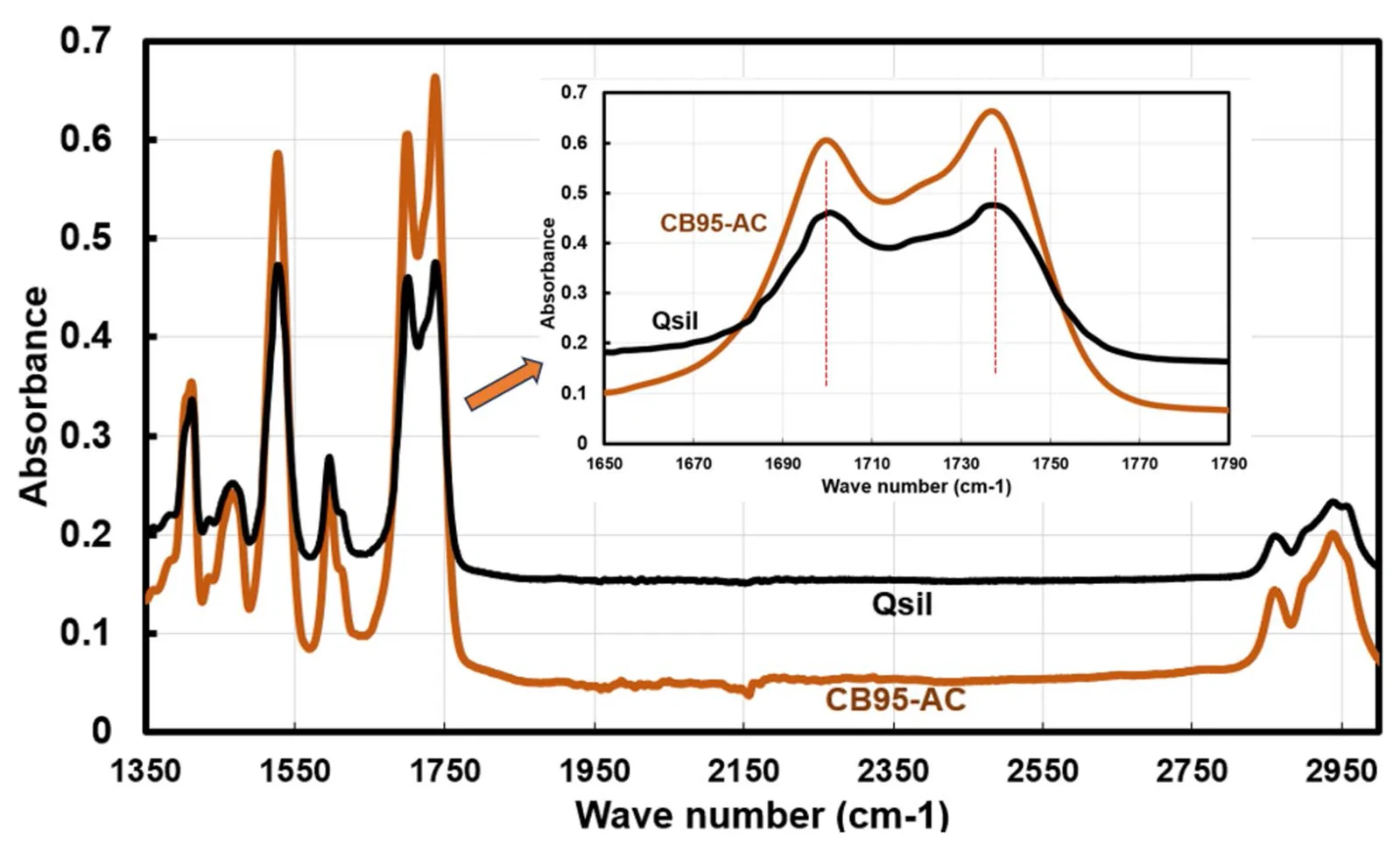
FTIR spectrum of TPU films: FTIR spectra illustrating the carbonyl absorption bands used to estimate relative hydrogen bonding. CB95-AC and Qsil showed comparable carbonyl peak ratios, indicating similar overall hard-segment hydrogen bonding by FTIR. A higher proportion of hydrogen-bonded carbonyls indicates greater hard-segment content and increased material hardness. The results demonstrate that CB95-AC and Qsil have similar degree of hard-segment hydrogen bonding

FTIR analysis (Fig. 3) showed similar carbonyl absorption characteristics for CB95-AC and Qsil. The ratios of strongly hydrogen-bonded to free carbonyl peak areas (1700/1735 cm^−1^) were 0.46 for CB95-AC and 0.48 for Qsil, indicating comparable hard-segment hydrogen bonding and microphase organization. No meaningful differences in overall carbonyl hydrogen bonding were detected between the two polymers.

The effect of H_2_O_2_ accelerated aging was further evaluated using the free-to-hydrogen-bonded carbonyl peakarea ratio, calculated as (A1735)/(A(1722) + A(1700)). This ratio decreased slightly after aging, from 0.61 to 0.57 for CB95-AC and from 0.57 to 0.54 for Qsil. These small decreases indicate a minor relative reduction in free or unassociated carbonyls compared with hydrogen-bonded carbonyls; however, the limited magnitude of the change does not indicate a substantial alteration in polymer microphase organization. Because FTIR provides only an indirect assessment of oxidation and microphase morphology, subtle chemical or domain-level changes cannot be excluded.

**Fig. 4:**
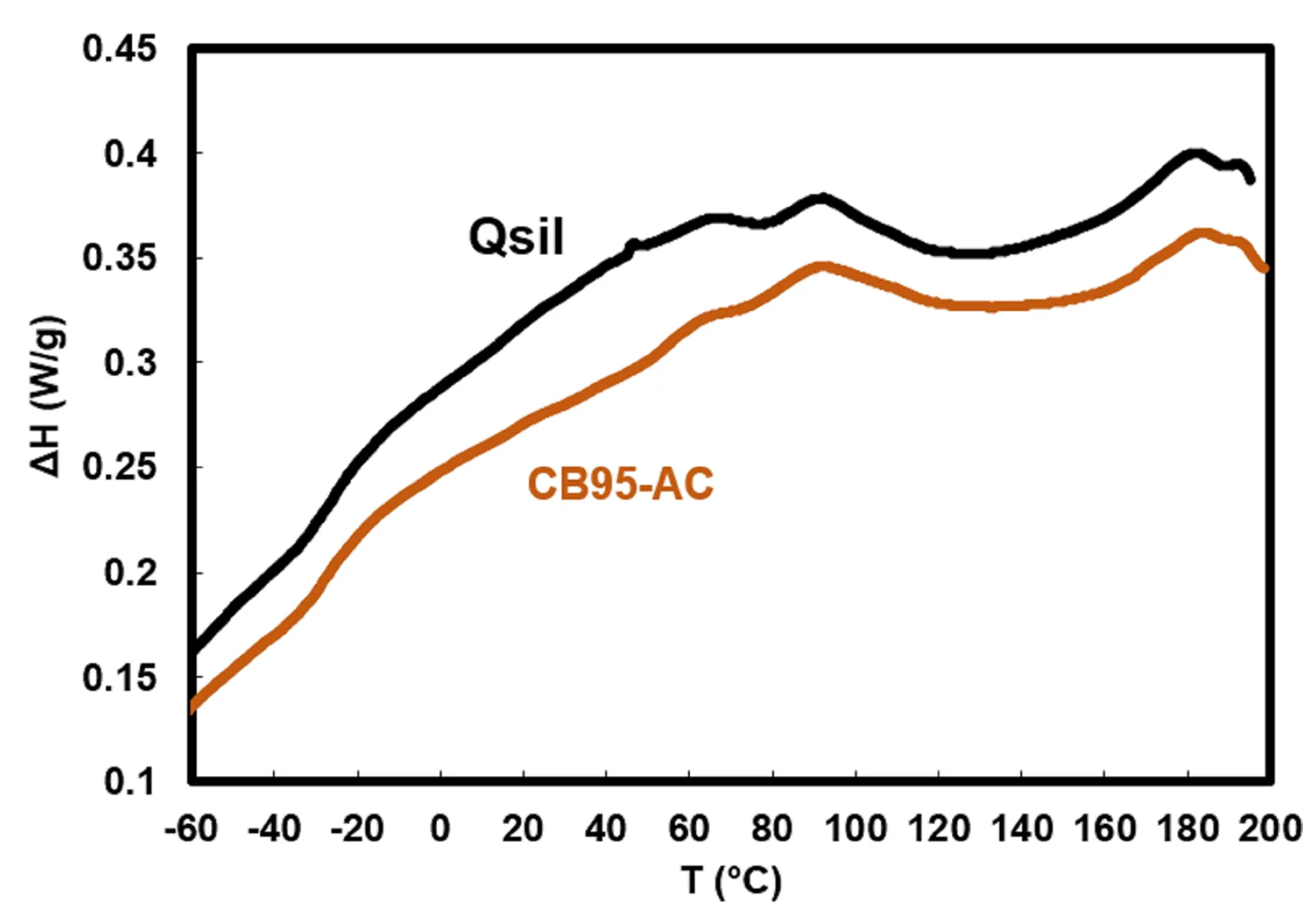
DSC first-heating thermograms of Qsil and CB95-AC TPU films. Both materials exhibited similar thermal responses over the evaluated temperature range, with comparable transition temperatures and broad thermal features characteristic of segmented thermoplastic polyurethanes. No major shifts in the positions of the observed transitions were detected, suggesting similar phase structure and microphase organization

The DSC first-heating thermograms (Fig. 4) of Qsil and CB95-AC exhibited broadly similar thermal transition behavior across the evaluated temperature range. Both materials showed comparable transition regions beginning near − 70 °C and broad thermal features characteristic of segmented polyurethane systems, suggesting similar phase-separated microstructures and hard/soft segment organization. No significant shifts in the transition regions were observed, indicating that incorporation of the siloxane-containing phase in Qsil did not substantially alter the bulk microstructure from a DSC perspective.

### Accelerated Wear Testing and Structural Durability

**Fig. 5:**
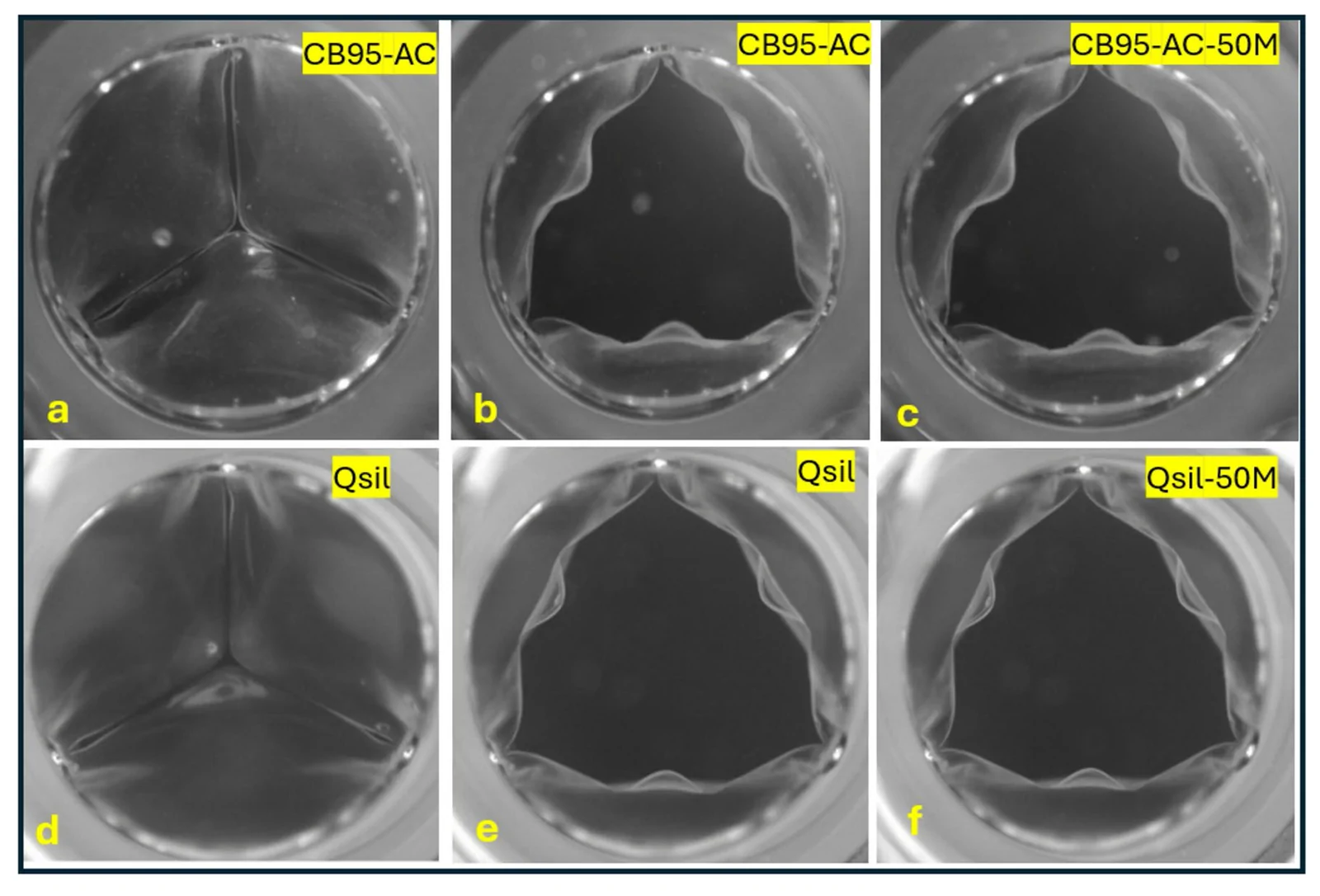
Representative images of valve closure and opening during accelerated wear testing (AWT). **a** CB95-AC in the closed position at *t* = 0; **b** CB95-AC in the open position at *t* = 0; **c** CB95-AC in the open position after 50 million cycles; **d** Qsil in the closed position at *t* = 0; **e** Qsil in the open position at *t* = 0; and **f** Qsil in the open position after 50 million cycles. Both valves exhibited good leaflet coaptation in the closed state and stable opening behavior in the open state. The open-orifice area was calculated from the valve opening images, and Qsil showed a slightly larger orifice area than CB95-AC. After 50 million cycles, no significant change in projected orifice area was observed for either valve, indicating that AWT did not noticeably alter valve opening performance

Representative valve configurations during AWT are shown in Fig. 5. Both CB95-AC and Qsil valves demonstrated symmetric trileaflet opening and effective leaflet coaptation in the closed position at baseline and after 50 million cycles. No visible structural damage, such as tearing, delamination, or gross deformation, was observed in either material following testing.

The projected orifice area was higher for Qsil than CB95-AC, with mean values of 329 ± 2 mm^2^ and 309 ± 22 mm^2^, respectively, corresponding to an approximately 6.5% increase in the Qsil group. However, statistical comparison using a Welch *t* test showed that the difference was not statistically significant (*p* > 0.05), indicating that the projected opening areas were comparable between the two valve groups. For one representative valve from each group, projected orifice area was also measured before and after 50 million cycles. CB95-AC showed minimal change from 314.5 mm^2^ at baseline to 315.5 mm^2^ after cycling, while Qsil changed from 327 mm^2^ to 318.5 mm^2^. These results suggest that 50 million cycles had a limited effect on projected orifice area under the tested conditions. It should be noted that AWT was performed under a moderate differential pressure of approximately 25–35 mmHg, and also valve casting-related factors, including mold geometry, dipping uniformity, and leaflet thickness, may contribute alongside material properties to the observed differences.

**Fig. 6:**
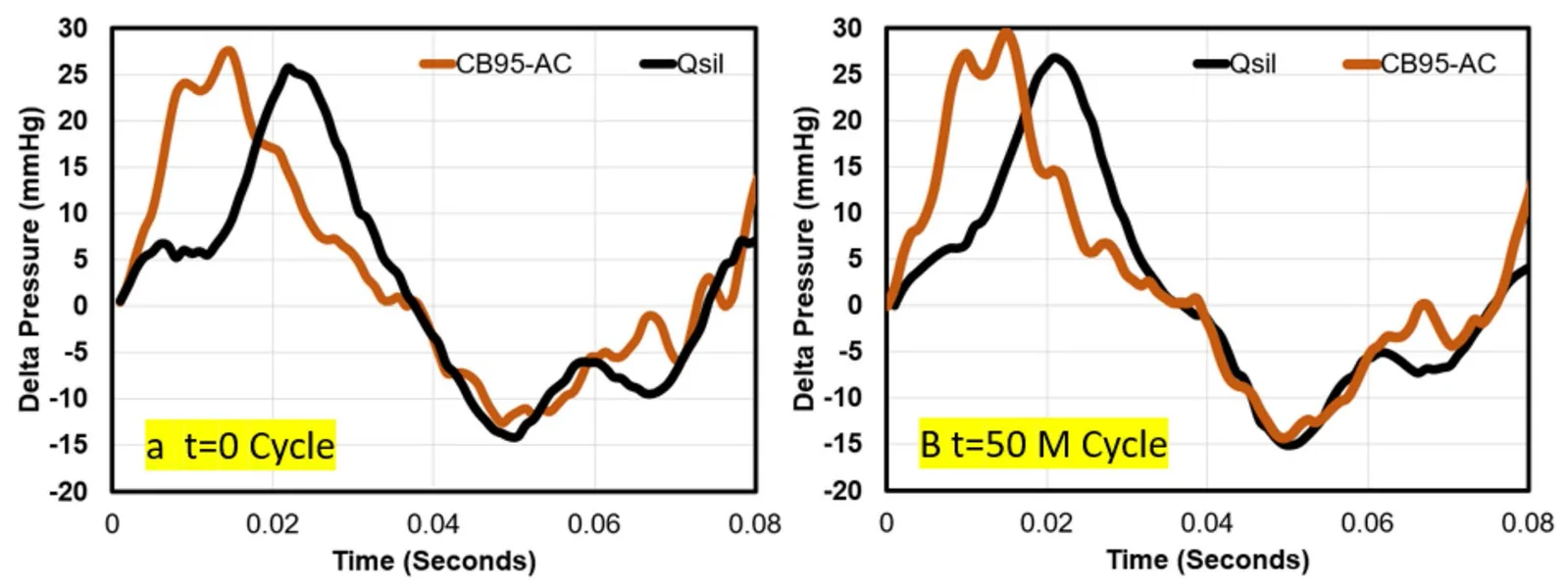
Differential pressure profiles of CB95-AC and Qsil valves recorded during accelerated wear testing (AWT) at **a** the initial cycle and **b** after 50 million cycles. The overall waveform shape remained similar after cycling for both materials, indicating stable pressure performance during AWT. CB95-AC exhibited an earlier and slightly sharper positive pressure peak, whereas Qsil showed a broader and slightly delayed peak, consistent with differences in dynamic response between the two valve materials

Differential pressure waveforms recorded during AWT are shown in Fig. 6. Both materials demonstrated stable pressure profiles over time, with minimal changes between baseline and 50 million cycles. CB95-AC exhibited an earlier and slightly higher positive pressure peak, indicating a faster dynamic response. In contrast, Qsil exhibited a delayed peak and a broader pressure profile, consistent with a more compliant and damp mechanical response. Both materials exhibited similar zero-crossing timing, indicating comparable overall cycle dynamics.

### Hemodynamic Performance and Flow Structure (PIV Analysis)

The experimental configuration for particle image velocimetry (PIV) measurements is shown in Fig. 1 [21–25]. Both valve types generated stable, physiologic pulsatile flow conditions, with peak inflow rates of approximately 5 L/min. The inflow and outflow waveforms demonstrated consistent cyclic behavior for both materials, indicating reliable valve operation under simulated left ventricular conditions.

**Fig. 8:**
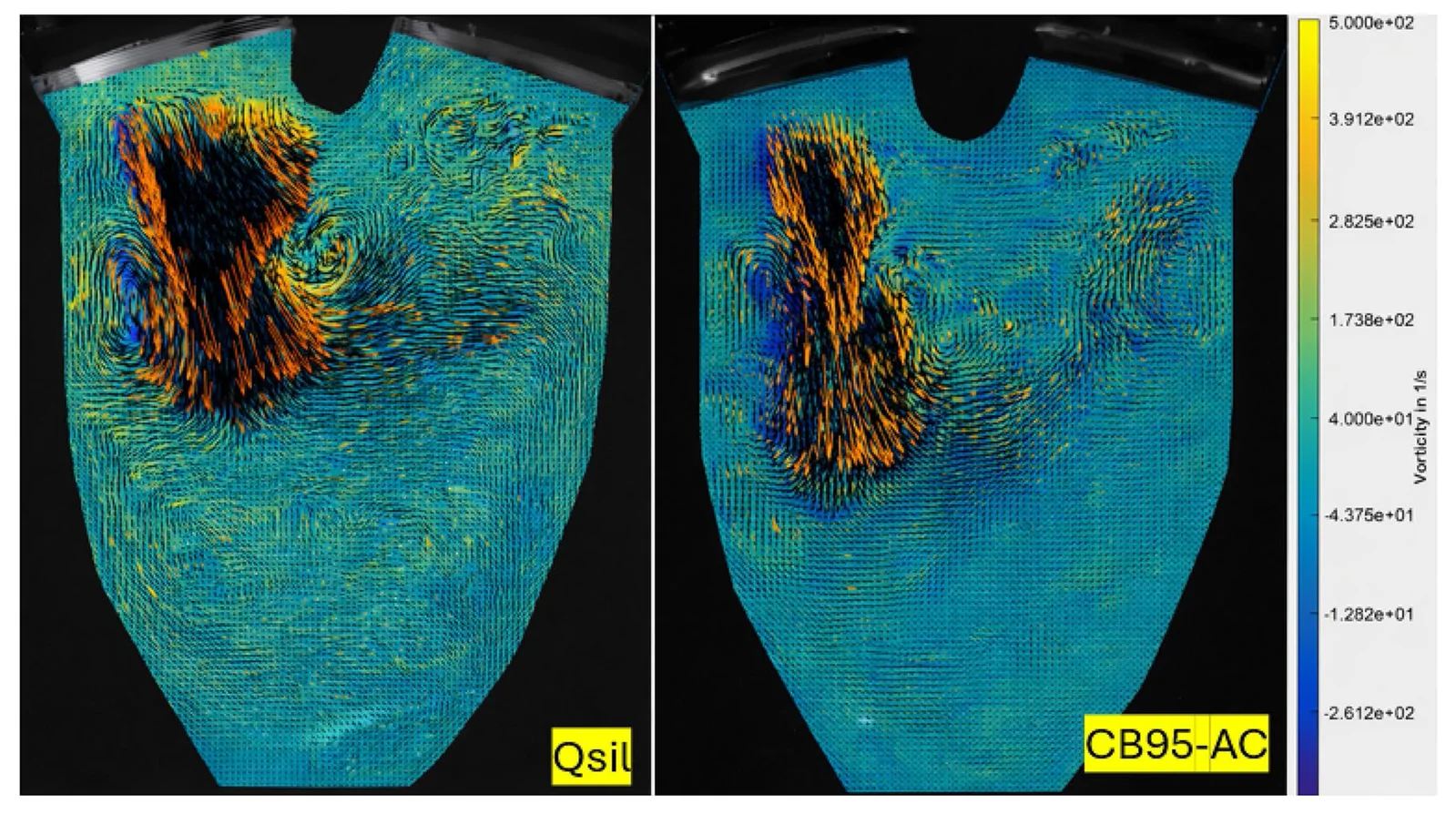
PIV-derived velocity vector fields and vorticity distribution in the left ventricle model for polymeric valves fabricated from Qsil (left) and CB95-AC (right). Both valves produced a central inflow jet accompanied by vortex formation within the ventricular chamber. However, differences in jet width and vortex distribution were observed between the two materials, with Qsil showing a broader jet and more extended recirculation regions, while CB95-AC exhibited a narrower jet and more localized vortical structures. The color scale indicates vorticity intensity and sign

Velocity field and vorticity analyses at peak diastole (Fig. 8) showed that both valve types generated a central inflow jet with associated vortex formation within the left ventricular chamber. Overall, the flow patterns were comparable, although subtle differences were observed. The Qsil valve produced a slightly broader and more spatially distributed high-vorticity region, suggesting more diffuse intraventricular recirculation and flow redirection, whereas the CB95-AC valve generated a narrower inflow jet with more localized vortical structures. The Qsil valve also showed a slightly more oscillatory pressure response, consistent with more compliant leaflet behavior. A small transient negative-flow signal was observed near valve closure for both valves, which may be associated with localized flow reversal during closure or measurement sensitivity near the flowmeter location. Review of the PIV velocity fields and flow visualization showed no visible reverse flow or leakage around the annular fixation region when the valve was fully closed, suggesting effective leaflet coaptation and no observable paravalvular leakage or significant regurgitation under the tested conditions.

**Fig. 9:**
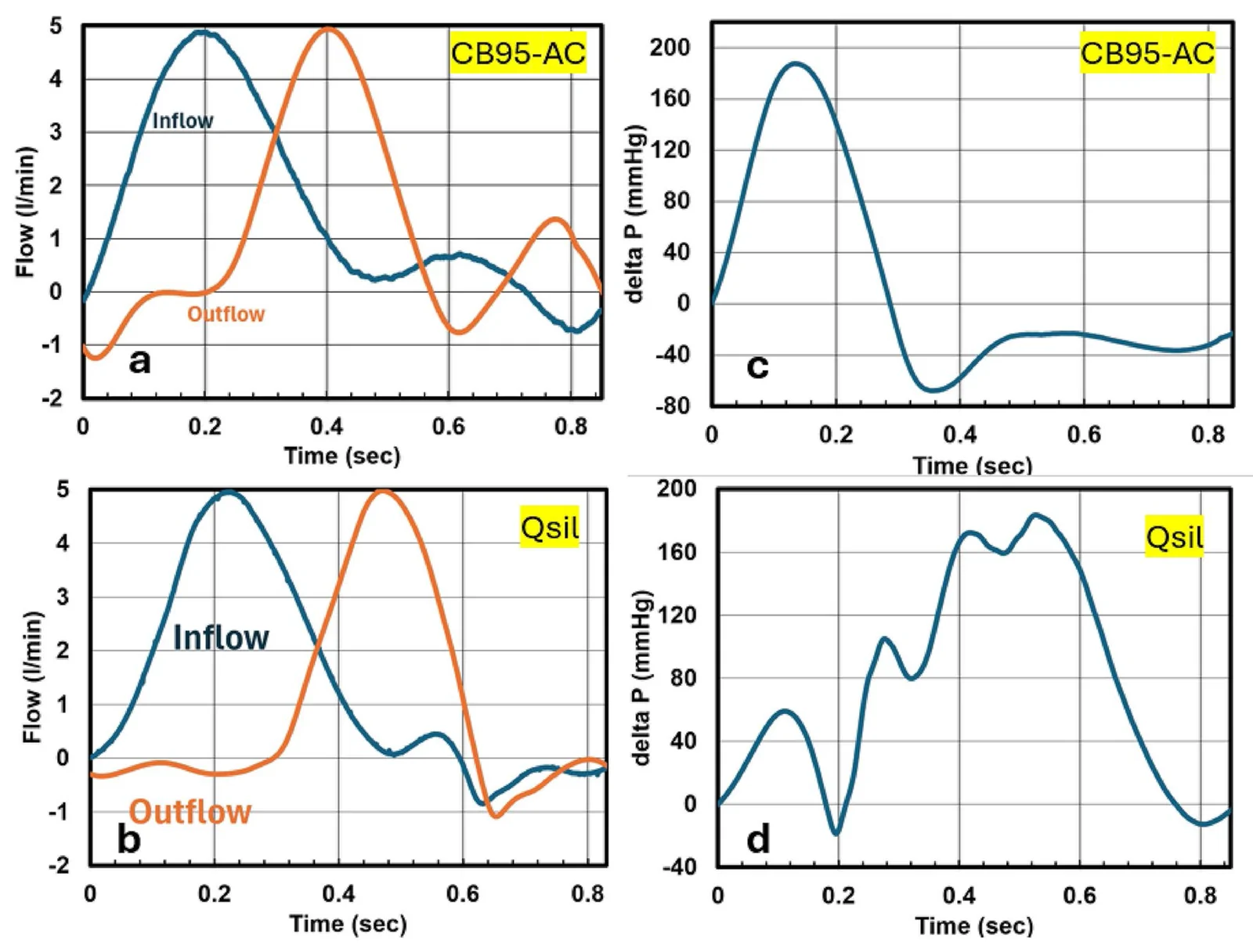
Pulsatile ventricular inflow and outflow waveforms and corresponding transvalvular pressure-differential (ΔP) profiles for polymeric mitral valves fabricated from CB95-AC and Qsil. Panels **(a)** and **(b)** show the ventricular inflow and outflow rate profiles for the CB95-AC and Qsil valves from flowmeters indicated in Fig.7, respectively, over one pulse duplicator cycle. Panels **(c)** and **(d)** present the corresponding transvalvular pressure-differential (ΔP) profiles. Both valve designs exhibited a dominant early diastolic inflow peak followed by a smaller late-cycle filling wave, together with the corresponding ventricular outflow and pressure transitions. Although the overall waveform characteristics were comparable, subtle differences in flow and pressure profiles were observed between the two materials, consistent with differences in leaflet compliance and valve opening/closing dynamics

The pulsatile inflow and outflow waveforms (Fig. 9 a,b) demonstrated stable cyclic hemodynamic behavior, with both valves exhibiting a dominant early inflow peak followed by a smaller late-cycle filling peak. The corresponding transvalvular pressure waveforms (Fig. 9 c,d) showed repeatable pressure transitions throughout the pulse-duplicator cycle. Analysis of the PIV data enabled identification of five sequential flow phases within each cycle. During early valve opening, a strong inflow jet rapidly entered the ventricular chamber and initiated vortex formation. This was followed by reduced inflow as the driving pressure gradient declined, resulting in slower passive filling. Subsequent valve closure redirected flow through the loop outflow pathway, accompanied by weakening or displacement of the previously formed vortical structures. A brief transition phase with minimal mitral inflow then occurred before reopening. Finally, late- cycle filling produced a smaller secondary inflow peak prior to the next cycle.

### Dynamic Mechanical Analysis

**Fig. 10:**
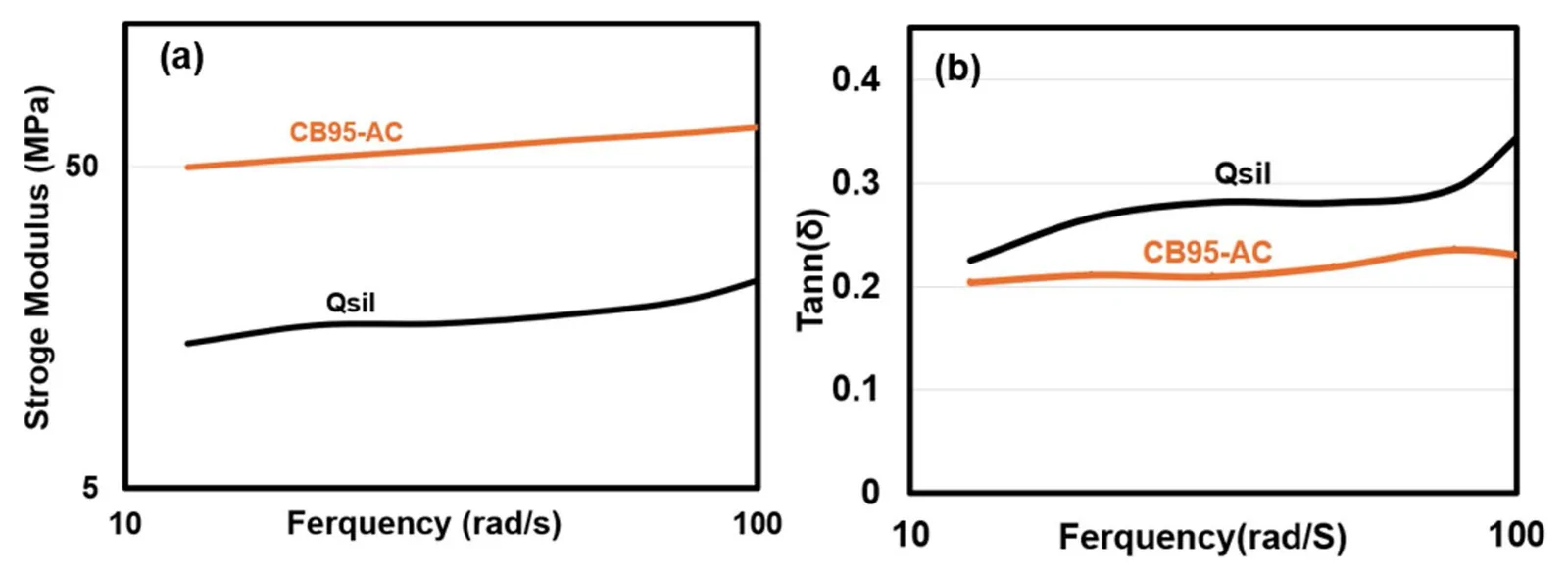
Dynamic mechanical analysis (DMA) of CB95-AC and Qsil. **a** Storage modulus measurements show that CB95-AC exhibits the higher stiffness, while Qsil displays lower moduli under low-amplitude strain (0.1%). **b** The tan δ curves indicate a lower energy dissipation for CB95-AC, reflecting its higher elasticity in small amplitude. A higher tan δ corresponds to increased energy dissipation within the material, highlighting differences in viscoelastic character among the TPU formulations

Dynamic mechanical analysis (Fig. 10) further highlighted the differences in the viscoelastic behavior of the two materials under low-amplitude strain deformation. CB95-AC exhibited a higher storage modulus across the tested frequency range, indicating greater stiffness. In contrast, Qsil exhibited a higher loss factor (tan δ), indicating increased energy dissipation and a more pronounced compliance response.

### In Vivo Functional Assessment

**Fig. 11:**
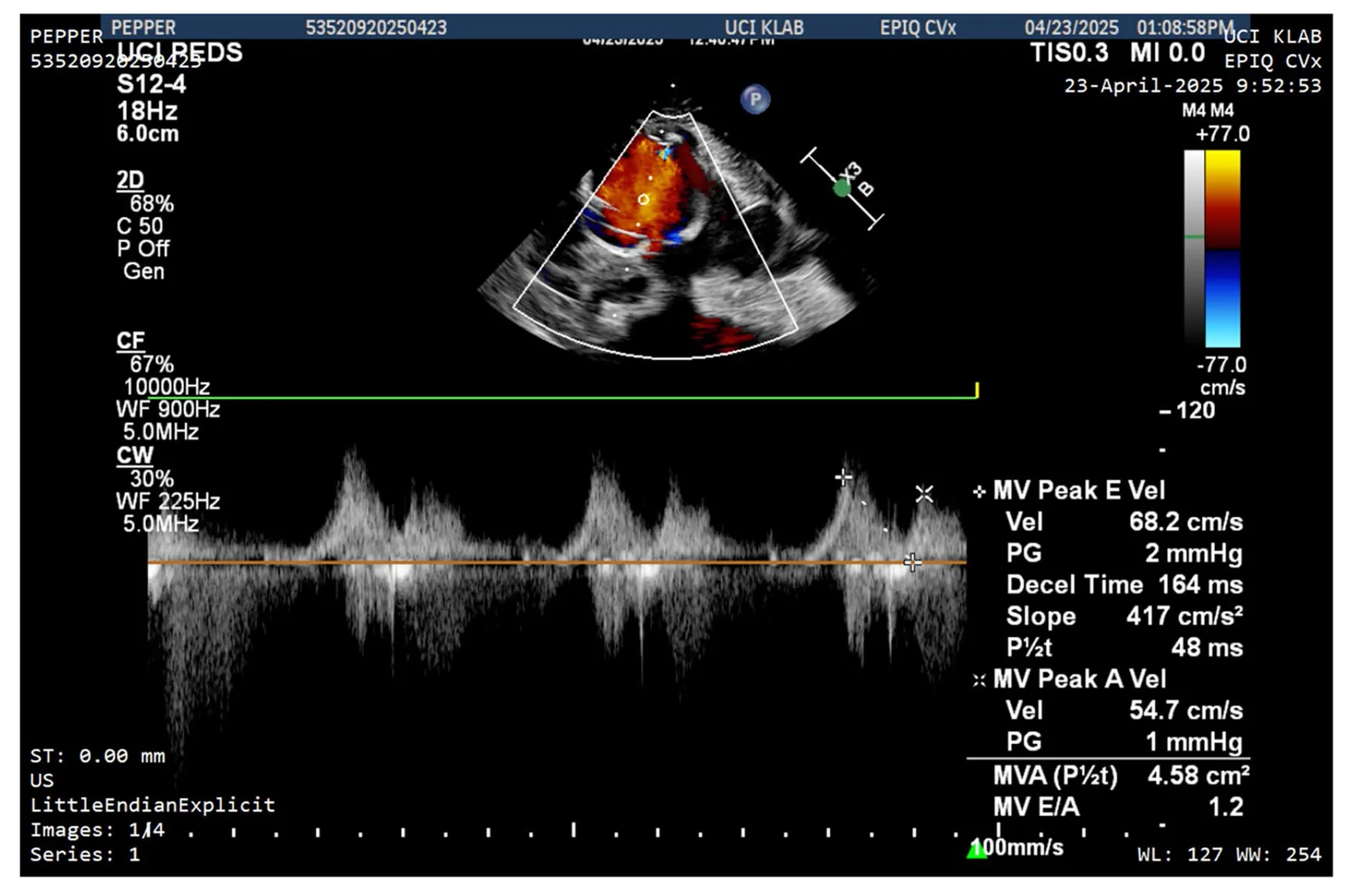
Representative Doppler echocardiographic assessment of transmitral inflow across the implanted polymeric mitral valve in the ovine model. The upper panel shows the color Doppler image of flow through the mitral valve, while the lower panel shows the corresponding continuous-wave Doppler waveform used to quantify diastolic filling parameters. The measured transmitral inflow values included a peak early filling velocity (E-wave) of 68.2 cm/s, a peak atrial filling velocity (A-wave) of 54.7 cm/s, and an E/A ratio of 1.2. Additional parameters included a peak gradient of 2 mmHg for the E-wave, 1 mmHg for the A-wave, a deceleration time of 164 ms, a deceleration slope of 417 cm/s^2^, a pressure half-time of 48 ms, and a calculated mitral valve area of 4.58 cm^2^. Overall, the Doppler image demonstrates forward transmitral flow with distinct early and late diastolic filling components, consistent with functional opening of the implanted polymeric valve

An initial *in vivo* evaluation was performed using a CB95-AC valve implanted in an ovine model. Doppler echocardiographic results are shown in Fig. 11. The transmitral inflow waveform demonstrated a clear biphasic filling pattern with distinct early (E-wave) and atrial (A-wave) components.

The peak E-wave velocity was 68.2 cm/s, and the peak A-wave velocity was 54.7 cm/s, resulting in an E/A ratio of 1.2, consistent with physiologic diastolic filling. The corresponding peak transvalvular pressure gradients were low, approximately 2 mmHg for the E-wave and 1 mmHg for the A-wave, indicating minimal resistance to forward flow.

Additional parameters included a deceleration time of 164 ms, pressure half-time of 48 ms, and a calculated mitral valve area of 4.58 cm^2^. These values are consistent with adequate leaflet opening and the absence of significant stenosis. Color Doppler imaging demonstrated forward transmitral flow without evidence of major turbulence in the imaged plane. Overall, these findings indicate that the implanted CB95-AC polymeric valve maintained effective opening behavior and supported stable, low-gradient diastolic inflow under *in vivo* conditions.

## Discussion

This study investigated the relationship between polymer composition, mechanical behavior, and valve-level performance through a multiscale evaluation of two polycarbonate-based polyurethane materials for polymeric mitral valve applications. Although CB95-AC and Qsil exhibited similar FTIR spectra and comparable DSC thermal transition behavior, differences in rheological and mechanical responses were associated with measurable differences in valve performance.

At the material scale, the rheological results shown in Fig. 2a indicate that Qsil exhibited higher viscosity and more pronounced shear-thinning behavior than CB95-AC, consistent with stronger intermolecular associations and reduced chain mobility in solution [26]. This behavior may also be influenced by the presence of siloxane segments, which have been reported to promote physical network formation and alter chain mobility [27]. In contrast, CB95-AC exhibited lower viscosity and more Newtonian-like behavior, suggesting more effective chain dissolution and reduced intermolecular or hard-segment association under the tested conditions. These rheological differences are directly relevant to dip molding, where the dependence of viscosity on shear rate can affect coating uniformity, leaflet thickness, and fabrication reproducibility. The stronger shear-rate-dependent viscosity observed for Qsil suggests that its dipmolding process may be more sensitive to parameters such as dipping speed, dwell time, withdrawal rate, and solution preparation. Since molecular weight and molecular-weight distribution can also influence solution viscosity and low-frequency elasticity, complementary GPC analysis of the same resin lots is in progress to further clarify the contribution of molecular-weight effects.

Mechanical testing demonstrated broadly similar tensile behavior for both materials. CB95-AC exhibited slightly higher tensile stress at low strain, whereas Qsil showed lower residual strain and improved recovery under cyclic and large-strain loading (Fig. 2b, c, e). Under physiologic conditions (wet, 37 °C; Fig. 2d), both materials showed comparable cyclic response behavior, with lower residual strain compared with the room-temperature condition. These behaviors can be interpreted in the context of segmented TPU microstructure, in which soft segments provide extensibility and hard segments act as physical crosslinks that stabilize the network [19]. Previous studies have shown that TPUs can undergo strain-induced microstructural reorganization, where hard domains fragment into smaller structures that may function as additional physical crosslinks and improve fatigue resistance [19], [28]. Although Qsil showed some improved strain recovery, FTIR and DSC results showed comparable bulk chemical and thermal features between the two materials. Therefore, the present study cannot directly confirm differences in hard-segment dispersion or microphase morphology, and the relationship between composition and cyclic recovery should be interpreted as associative rather than strictly mechanistic. Although consistent trends were observed across material characterization, mechanical testing, and valve-level evaluation, the present study does not directly establish a causal chemistry-to-function pathway. Techniques such as GPC, SAXS, AFM phase imaging, or other microstructural characterization methods would provide additional insight into molecular-weight effects, microphase morphology, and hard-segment organization, thereby strengthening mechanistic interpretation.

The somewhat higher tear resistance observed for CB95-AC is likely related to its higher hardness and greater resistance to crack initiation and propagation [17]. For segmented TPUs, tear and tensile responses are strongly influenced by hard/soft segment interactions, intermolecular association, and material hardness. Overall, the mechanical results suggest that CB95-AC provides greater stiffness and tear resistance, while Qsil provides improved strain recovery and compliance. This balance is important for polymeric valve design, where the leaflet material must resist tearing and fatigue while maintaining sufficient flexibility for repeated opening and closure.

Environmental exposure further highlighted the sensitivity of TPU behavior to physiologic conditions. Moisture reduced tear resistance in both materials, consistent with water-induced plasticization, disruption of hydrogen bonding, and increased chain mobility; however, moisture also improved strain recovery during cyclic loading. Oxidative aging reduced tear resistance, consistent with previously reported degradation mechanisms in polycarbonate urethanes [29]. Prior literature indicates that polycarbonatebased urethanes and polysiloxane-based urethanes exhibit strong biostability and weathering resistance among medical-grade TPUs [29], [30].

At the valve level, both materials demonstrated stable function during accelerated cyclic wear testing. The AWT results shown in Fig. 5 indicate that both valve designs maintained structural integrity after 50 million cycles, with no visible tearing, leaflet fracture, delamination, major deformation, or loss of coaptation. Similar stability in polymeric valves has been reported in prior durability studies, where retention of flow and pressure characteristics is considered an important indicator of functional performance [4]. In the present study, the AWT was performed as a comparative durability screen under identical test conditions rather than as a formal ISO 5840 qualification study, because the peak measured differential pressure was approximately 25–30 mmHg. Within this comparative framework, both materials maintained stable cyclic behavior and valve integrity.

Differences in pressure response were consistent with the material-level mechanical behavior. CB95-AC exhibited a slightly sharper pressure response, consistent with a stiffer leaflet response, whereas Qsil showed a broader and more damped profile, suggesting slightly greater compliance. This interpretation is supported by DMA results, where Qsil exhibited a lower storage modulus than CB95-AC in the small-amplitude deformation range. A lower storage modulus indicates reduced elastic stiffness, which may contribute to the larger opening and broader pressure response observed for Qsil. The siloxane-containing segments in Qsil may contribute to lower modulus, higher damping, and improved strain recovery by increasing chain flexibility and energy dissipation. In contrast, the higher storage modulus of CB95-AC is consistent with its faster dynamic response, narrower pressure peak, and higher tear resistance.

The projected orifice area was slightly larger for Qsil than for CB95-AC, with mean values of 329 ± 2 mm^2^ and 309 ± 22 mm^2^, respectively. This corresponded to an approximately 6.5% increase in the Qsil group; however, the difference was not statistically significant (p > 0.05), indicating that the projected opening areas were comparable between the two valve groups. The slightly larger opening area of Qsil may be related to its greater compliance, although fabrication-related factors such as leaflet thickness, mold geometry, dip-molding uniformity, and casting variability may also contribute. For one representative valve from each group, projected orifice area was also measured before and after 50 million cycles. CB95-AC showed minimal change from 314.5 mm^2^ at baseline to 315.5 mm^2^ after cycling, while Qsil changed from 327 mm^2^ to 318.5 mm^2^. These results suggest that 50 million cycles had a limited effect on projected orifice area under the tested conditions.

PIV analysis demonstrated physiologic inflow patterns with vortex formation for both valve designs. The vorticity differences calculated from PIV (Fig. 8a,b) showed that both valves generated central inflow jets with associated intraventricular vortex formation, confirming effective diastolic flow behavior. However, Qsil produced a broader and more spatially distributed vorticity field, whereas CB95-AC generated a narrower inflow jet with more localized vortical structures. These differences may be associated with leaflet compliance and projected orifice area. The relationship between leaflet mechanics and flow organization has been described previously, where leaflet geometry and mechanical properties influence jet development and intraventricular flow patterns in polymeric valves [12]. Although that work focused on aortic configurations, the same general principles are relevant to mitral inflow.

The organization of intraventricular flow is functionally important because efficient vortex formation has been linked to ventricular filling, momentum preservation, and reduced energy dissipation [26–30]. In this context, the broader vortex structures observed with Qsil may reflect more diffuse intraventricular recirculation and flow redirection, while the more localized jet observed with CB95-AC is consistent with a slightly stiffer leaflet response. The observed PIV flow patterns and AWT pressure waveforms are consistent with the film-level viscoelastic differences; however, they should be interpreted as associative rather than strictly causal. Leaflet dynamics are governed by coupled fluid–structure interactions, including modulus, hysteresis, thickness, bending stiffness, damping, geometry, and pressure loading.

A small transient negative-flow signal was observed near valve closure for both valves. This may be associated with localized flow reversal during leaflet coaptation or measurement sensitivity near the flowmeter location, rather than a material limitation [12]. Importantly, review of the PIV velocity fields and flow visualization showed no visible regurgitant flow, backflow, or paravalvular leakage around the annular fixation region when the valves were fully closed, suggesting effective leaflet coaptation and stable sealing under the tested in vitro conditions. Future studies will further characterize sealing performance using quantitative PVL metrics, including regurgitant volume, regurgitant fraction, leakage volume, and Doppler-based assessment in flow loop and in vivo models.

Preliminary in vivo evaluation of the CB95-AC valve (Fig. 11) demonstrated preserved transmitral inflow with a physiologic E/A ratio, low transvalvular gradients, and no evidence of significant obstruction. These findings are consistent with established echocardiographic interpretation of diastolic function [26] and support the ability of the polymeric valve scaffold to reproduce physiologic filling behavior in an acute animal model. The observed flow behavior is also consistent with prior studies demonstrating that appropriately tuned leaflet mechanics can restore physiologic intraventricular flow patterns [28]. The projected geometric orifice area of the CB95-AC valve measured from fully open in vitro images was 309 ± 22 mm^2^ (3.09 ± 0.22 cm^2^). In comparison, the in vivo echocardiographic assessment yielded a mitral valve area of 4.58 cm^2^ based on the pressure half-time method. These values represent different measurements, as the projected geometric area is a two-dimensional image-based opening area, whereas the echocardiographic mitral valve area reflects functional valve opening under physiologic in vivo conditions.

In addition to the material-focused evaluation presented here, a separate ongoing study is investigating the influence of valve manufacturing parameters on leaflet geometry and valve performance. This work includes systematic evaluation of dip-molding variables such as polymer solution viscosity, dipping speed, dwell time, withdrawal rate, drying conditions, mold design and geometry, and leaflet thickness. A design-of-experiments (DOE) approach is being employed to quantify the relative contribution of each processing parameter and to establish manufacturing–structure–function relationships. These studies are expected to provide further insight into how fabrication variables influence valve mechanics, leaflet kinematics, hydrodynamic performance, and long-term durability, complementing the material-comparison results reported in the present work.

Several limitations should be considered: Mw/MWD may have contributed to the observed rheological differences between CB95-AC and Qsil. To further strengthen this interpretation, a validated GPC method is being developed using LiBr-containing solvent systems to improve reproducibility and better distinguish molecular-weight effects from chemistry-dependent effects.

The PIV measurements were performed in a single two-dimensional plane and therefore did not capture out-of-plane velocity components or fully resolve three-dimensional vortex structures. Future stereo-PIV or volumetric/tomographic PIV studies would allow assessment of out-of-plane velocity components, vortex-ring asymmetry, and three-dimensional flow organization. Second, the accelerated cyclic wear testing was used as a comparative durability screen and did not meet the differential pressure requirements for formal ISO 5840 mitral durability qualification. Full mitral-position durability evaluation will require extended cycling, appropriate closing differential pressure, defined regurgitant acceptance criteria, and intermediate hydrodynamic reassessment consistent with ISO 5840-3.

Additional limitations relate to fabrication and mechanical characterization. Dip-molding parameters such as solvent evaporation rate, mandrel surface, drying schedule, ambient conditions, and withdrawal uniformity may contribute to local thickness variation and through-thickness microstructural organization. Bulk tensile and DMA measurements provide useful insight into intrinsic polymer behavior and allow direct comparison between formulations; however, valve motion is also influenced by leaflet-scale factors including local thickness, bending stiffness, possible in-plane anisotropy, residual stresses, and processing-related morphology. Future work incorporating local leaflet thickness mapping, high-speed leaflet imaging, and leaflet-scale mechanical testing, such as bending and biaxial testing, would further strengthen the connection between film properties and valve dynamics.

The PIV and AWT results should be interpreted as correlative rather than fully causal, as direct leaflet kinematics were not measured during valve operation. To address this, a separate study is underway using fluorescent tracer particles embedded in the leaflets for high-speed imaging and quantitative leaflet motion tracking. In parallel, a two-way ANSYS fluid–structure interaction model is being developed to simulate coupled leaflet deformation and flow dynamics.

The in vivo evaluation was limited to a single acute implantation of a CB95-AC valve and therefore does not address chronic performance, remodeling, thrombogenicity, hemocompatibility, calcification, or long-term durability. Hemocompatibility remains essential for translation of polymeric and bio-hybrid heart valves. Although the present study focused on mechanical behavior, hydrodynamic performance, durability screening, and acute in vivo feasibility, platelet activation, hemolysis, thrombosis, complement activation, and protein adsorption require dedicated evaluation. These endpoints are being addressed in a separate hemocompatibility-focused study of related polymeric valve constructs. Additional chronic large-animal studies with explant histology and SEM will further define long-term blood-contact performance and bio-hybrid integration.

Long-term polymeric valve performance may also be influenced by coupled degradation mechanisms such as oxidative degradation, hydrolytic chain scission, fatigue-related leaflet thinning, surface defect propagation, stress corrosion cracking, and calcification. While the CoCl_2_/H_2_O_2_ protocol used here provides an accelerated oxidative challenge, it does not directly assess calcification or fully reproduce clinically relevant long-term degradation. Dedicated calcification testing, including calcium-phosphate exposure or subdermal implantation with quantitative Ca/P analysis and histologic staining, will be important in future studies and is being addressed in a separate work focused on calcification of related polymeric valve constructs. Future translation of polymeric mitral valve constructs would also require comprehensive regulatory testing, including biocompatibility, chemical characterization, extractables/leachables, hemocompatibility, ISO 5840-3 durability, chronic large-animal studies, and clinical evaluation.

In conclusion, this study indicates that material-dependent differences in polyurethane composition and viscoelastic response were associated with differences in mechanical behavior, valve durability, and flow characteristics. Qsil exhibited a more compliant response with improved strain recovery and a slightly larger projected orifice area, whereas CB95-AC provided greater stiffness and tear resistance. Despite these differences, both valve designs maintained structural integrity and stable function after 50 million cycles of accelerated cyclic wear testing and generated physiologic flow patterns during PIV evaluation. Preliminary acute in vivo assessment of the CB95-AC valve further demonstrated successful implantation and preserved transmitral function with low pressure gradients. These findings support the continued development of polycarbonate-based polyurethane materials for polymeric mitral valve applications and underscore the importance of tailoring material properties to achieve durable and physiologic valve performance.

## Figures and Tables

**Fig.7:**
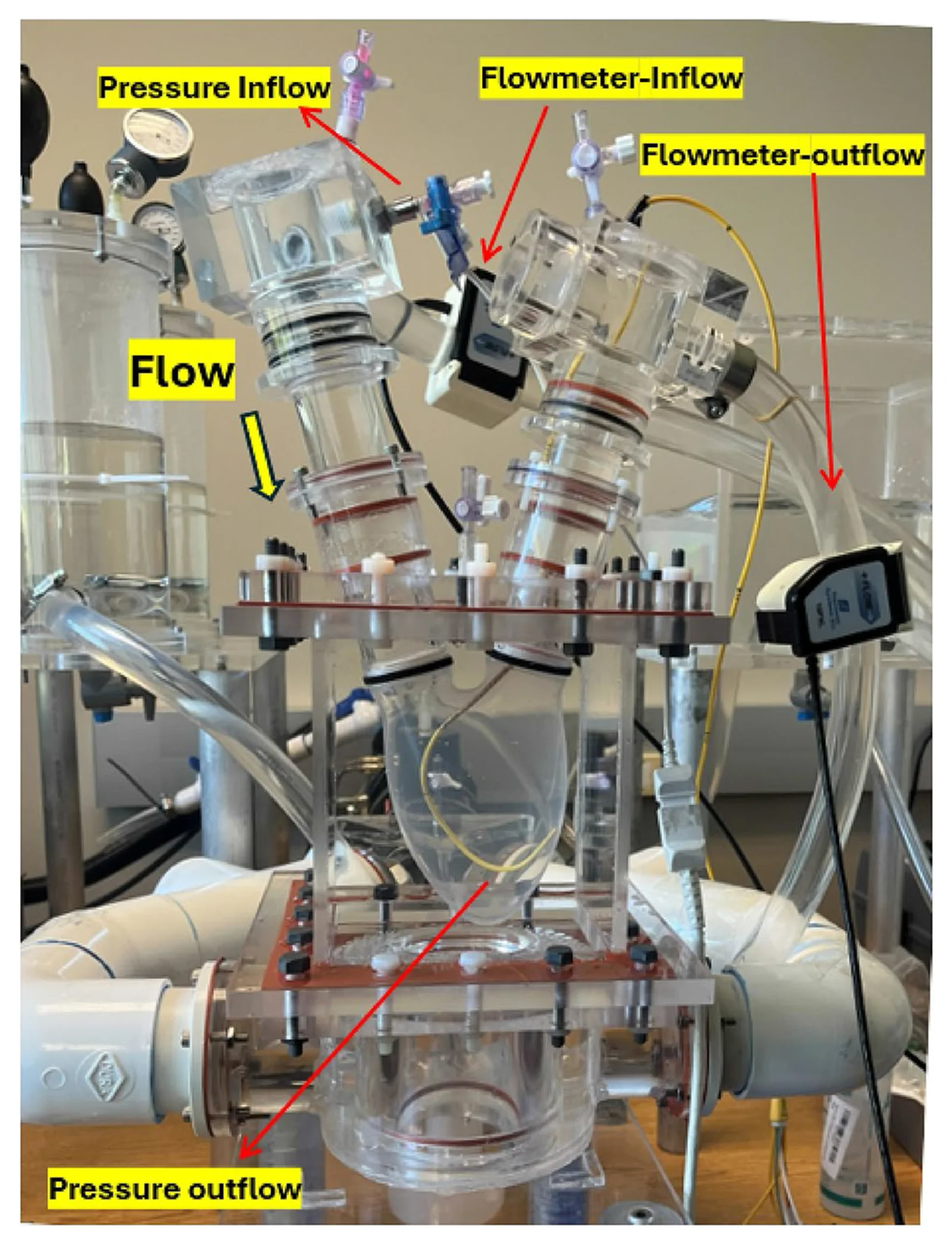
Experimental heart-flow loop used for valve hydrodynamic testing and particle image velocimetry (PIV) measurements. The setup includes the test chamber containing the polymeric valve, inflow and outflow flowmeters, and pressure measurement ports located upstream and downstream of the valve. The direction of flow through the system is indicated by the arrow. This configuration was used to monitor pressure and flow conditions across the valve during pulsatile testing and to correlate valve motion with the measured hemodynamic response

## Data Availability

The data underlying this article will be shared by any reasonable request to the corresponding author.
